# Prioritization of pathogenic mutations in the protein kinase superfamily

**DOI:** 10.1186/1471-2164-13-S4-S3

**Published:** 2012-06-18

**Authors:** Jose MG Izarzugaza, Angela del Pozo, Miguel Vazquez, Alfonso Valencia

**Affiliations:** 1 Structural Biology and BioComputing Programme, Spanish National Cancer Research Centre (CNIO), Madrid, Spain

## Abstract

**Background:**

Most of the many mutations described in human protein kinases are tolerated without significant disruption of the corresponding structures or molecular functions, while some of them have been associated to a variety of human diseases, including cancer. In the last decade, a plethora of computational methods to predict the effect of missense single-nucleotide variants (SNVs) have been developed. Still, current high-throughput sequencing efforts and the concomitant need for massive interpretation of protein sequence variants will demand for more efficient and/or accurate computational methods in the forthcoming years.

**Results:**

We present KinMut, a support vector machine (SVM) approach, to identify pathogenic mutations in the protein kinase superfamily. KinMut relays on a combination of sequence-derived features that describe mutations at different levels: (1) Gene level: membership to a specific group in Kinbase and the annotation with GO terms; (2) Domain level: annotated PFAM domains; and (3) Residue level: physicochemical features of amino acids, specificity determining positions, and functional annotations from SwissProt and FireDB. The system has been trained with the set of 3492 human kinase mutations in UniProt for which experimental validation of their pathogenic or neutral character exists. In addition, we discuss the relative importance of these independent properties and their combination for the development of a kinase-specific predictor. Finally, we compare KinMut with other state-of-the-art prediction methods.

**Conclusions:**

Family-specific features appear among the most discriminative information sources, which allow us to produce accurate results in a reliable and very simple way with minimal supervision. Our study aims to broaden the knowledge on the mechanisms by which mutations in the human kinome contribute to disease with a particular focus in cancer. The classifier as well as further documentation is available at http://kinmut.bioinfo.cnio.es/.

## Background

Current high-throughput resequencing screenings [1-3] represent a powerful set of techniques to discover large numbers of mutations. Of these, only a small fraction are causally implicated in disease onset and therefore, separating the wheat from the chaff is still a major challenge [4]. For a small subset of the new mutations discovered, experimental information is available regarding the relationship between the mutation and disease, and for an even smaller number of cases the underlying biochemical mechanism is known. However, there is no information for the remaining mutations. The requirement of a lot of resources implies that it is not feasible to experimentally test the association of all these mutations to disease, and to characterize their functional effects. Nevertheless, this problem is very amenable to *in silico* predictors [4-6]. Different approaches are currently available to predict the probability of a newly discovered mutation being implicated in disease. Some methods make use of several features to highlight crucial positions in a given protein, and hence, rules are derived to predict the pathogenicity of mutations. Another group of methods assumes that evolutionarily conserved protein residues are important for protein structure, folding and function, whereby mutations in these residues are considered deleterious [7]. Variations on this principle lead to methods that predict deleterious mutations by evaluating changes in evolutionarily conserved PFAM motifs [8]. Moreover, a number of systems use protein structures to characterize substitutions that significantly destabilize the folded state. There are also methods that integrate prior knowledge in the form of both sequence-based and structure-based features from a set of mutations (previously characterized as pathogenic or neutral) to train an automatic machine learning system. After this training process, the system can infer the pathogenicity of new mutations based on the knowledge acquired. These approaches, albeit similar in purpose, implement very different machine-learning methods. Among them, probably the most popular ones are: rule-based systems [9-11], decision trees [12], random forests [13,14], neural networks [15,16], Bayesian methods [17] and SVMs [18-21]. Recently, some meta approaches have been implemented, for instance, Condel [22] integrates five of the most widely employed computational tools for sorting missense single nucleotide variations.

Moreover, diverse datasets of mutations have been employed for benchmarking the performance of different methods, and depending on the scope of the predictor differences exist, as well. Most of the predictors are generally applicable to amino acid sequences from any protein family, while a few of them include properties that apply only to a given protein family of interest; i.e. protein kinase-specific predictors [19]. These family-related features bring discriminative information that justifies the development of specialized predictors.

A broad number of mutations in the protein kinase superfamily have been reported in the literature [23] and a subset of them is known to disrupt protein structure and function [24]. For some cases, since human protein kinases are involved in a plethora of physiological functions, this disruption can be causally associated to disease [25]. Still, the majority of protein kinase mutations are tolerated without apparent significant effects [26,27]. In previous publications, we have discussed the preferential distribution of germline pathogenic deviations [28] and driver somatic mutations [29] to regions of functional and structural importance. Here, we present the basis for the development of a computational method to predict the impact of mutations on the function of protein kinases - KinMut - based on these features.

In the work presented here, we explored the significance of disease-associated mutations in terms of sequence-derived characteristics at different levels:

1. *At the gene level:* membership to a KinBase group [30] and Gene Ontology [31] terms.

2. *At the domain level:* the occurrence of the mutation inside a PFAM [32] domain.

3. *At the residue level:* several properties including amino acid types, functional annotations from SwissProt [33] and FireDB [34], and specificity-determining positions (SDPs) [35].

Accordingly, we analyzed the independent significance of these properties and their combination with a support vector machine (SVM) and we discussed the benefits and pitfalls of using the information available for the development of a family-specific predictor. Finally, we compared KinMut with regard to other state-of-the-art prediction methods.

## Results

### Construction of the disease and neutral datasets

The method was trained and evaluated using a dataset derived from UniProt [33], which has been benchmarked previously for a number of classifiers with satisfactory results [36]. After our filtering pipeline, 865 mutations in 65 human kinases formed the ‘disease dataset’, whereas the ‘neutral dataset’ consisted of 2,627 mutations in 447 human kinases. For classification purposes, each mutation is described by 142 sequence-features. Full details in Methods.

### Optimization of the prediction method

To classify the mutations in the human kinome as disease-associated or neutral according to the sequence features of the mutations, we used a Support Vector Machine (SVM). This type of approach has previously been widely used to automatically prioritize disease-associated mutations [18,19,21,37,38] and it has been demonstrated to outperform other approaches such as Bayesian classifiers and neural networks [19].

Our implementation of the SVM relied on a radial basis function (rbf) kernel. Two parameters are crucial for the performance of the classifier, the soft-margin penalty (*C*) and the radius (γ): *C* represents the amount of errors allowed during the training and evaluation steps, while γ represents the width of the SVM radial function. These parameters can be optimized to improve the predictions. Hence, we conducted a grid search in a wide range of values for these parameters, to decide which pair predicts with the best performance, using the f-score as optimization function (a more detailed description of the optimization can be found in the Methods section). The optimal values used during the analyses corresponded to *C* = 8 and γ = 6 · 10^-4^ (Additional file 1). Comparable results were obtained when the under the ROC curve (AUC) was tested as selection criteria (Additional file 2).

### Evaluation of the performance of the classifier

We avoided over-fitting the classifier by applying a 10-fold cross-validation approach where 8 random sets were used during the training step, one for the validation phase and one for the evaluation. This process was repeated 90 times to allow all possible combinations of sets to be used during the evaluation and validation phases. Although the optimization of the kernel relies on the f-score, the performance of the classifier is assessed by several additional measures, such as accuracy, precision, recall and the Matthew’s correlation coefficient (MCC).

On average, the classifier predicted the pathogenicity of kinase mutations robustly (AUC=0.81). However, different threshold values could modulate the output of the classifier, as summarized in Table 1, and selecting an appropriate threshold is a critical step in developing a classifier. Relaxed thresholds, such as –0.75, enable the detection of more disease-associated mutations (increased recall), albeit at the cost of a larger number of false positives (reduced precision). Conversely, higher thresholds of conservative classifiers, such as –0.5, reduce the frequency of a mutation being classified as pathogenic, consequently predicting a smaller set of more reliable disease-associated mutations. We chose the threshold of –0.5 whereby the f-score (66.7%) is maximal. Using this conservative threshold, the classifier predicted 83.3% of the mutations correctly. Regarding the pathogenic dataset, 75.2% of the observed mutations were recovered on average across all k-folds with a precision of 60%. The average MCC was 0.6.

**Table 1:** Performance of the classifier depending on the SVM classification thresholds applied using all kinase groups

SVM threshold	Accuracy (%)	Precision (%)	**Recall** (%)	F-score	MCC
-1.00	74.3	46.5	89.1	61.1	0.6
-0.75	80.9	56.1	79.8	65.9	0.6
**-0.5**	**83.3**	**60.0**	**75.2**	**66.7**	**0.6**
-0.25	80.6	60.0	66.1	62.9	0.5
0.00	82.3	58.6	47.2	52.3	0.4

Performance of the classifier depending on the SVM classification thresholds applied using all kinase groups.

### Evaluation of the dependence of the performance on the abundance of information

When the different groups in which the protein kinase superfamily is divided were superimposed, we observed differences in the number of mutations that populated the groups (Table 2). These differences are consistent with the phylogenetic distribution of the literature-extracted mutations we observed earlier [23]. A small number of these groups contain most of the mutations, while others lack or contain very few disease-associated mutations. For the mutations in these less populated groups, only group membership suffices to consider them as neutral and this neutrality is likely an artifact due to the lack of experiments assessing the pathogenicity of the mutations.

**Table 2:** Number of mutations in each of the groups in which UniProt divides the protein kinase superfamily

Group	Disease	Neutral	Total
TK †	496	565	1061
TKL †	172	151	323
Atypical_PI3-PI4 †	49	138	187
CAMK †	40	518	558
Other	36	411	447
RGC †	23	35	58
CMGC †	18	178	196
AGC †	16	190	206
STE	7	222	229
Atypical_ADCK	6	14	20
Atypical_Alpha-type	1	88	89
CK1	1	52	53
NEK	0	45	45
Atypical_RIO	0	14	14
Atypical_PDK-BCKDK	0	5	5
Atypical_FAST	0	1	1

Number of mutations in each of the groups in which UniProt divides the protein kinase superfamily. The groups enriched in disease-associated mutations are highlighted by †.

Consequently, we evaluated the dependence of the results on the amount of disease-associated mutations available. A second dataset was generated with only the highly populated groups: TK, TKL, Atypical_PI3-PI4, CAMK, RGC, CMGC, AGC and Atypical_ADCK. Under this constraint the ‘disease dataset’ consisted of 814 mutations in 54 human kinases, while the neutral dataset contained 1,775 in 297 proteins.

When only the groups sufficiently populated with disease-associated mutations were considered, on average we correctly predicted 76.8% of the remaining mutations across all k-folds, with the optimized values of *C* = 8 and γ = 10^–4^ (Additional file 3). With respect to the pathogenic dataset, we recovered 73.3% of the disease-associated mutations with a precision of 64.7% (MCC: 0.5, Table 3), comparable to that obtained when all the mutations from all the kinase groups were considered, thereby confirming that the bias in the data does not significantly affect the results.

**Table 3:** Performance of the classifier depending on the SVM classification thresholds applied when using groups highly populated in disease mutations only

SVM threshold	Accuracy (%)	Precision (%)	Recall (%)	MCC
-1.000	71.5	51.4	88.9	0.6
-0.750	77.0	61.7	81.5	0.6
**-0.500**	**76.8**	**64.7**	**73.3**	**0.5**
-0.250	79.4	68.1	69.9	0.5
0.000	71.6	60.7	56.3	0.4

Performance of the classifier depending on the SVM classification thresholds applied when using groups highly populated in disease mutations only.

However, clear differences were observed when the groups were compared individually (Table 4). For the groups with a reasonable number of mutations, the performance of the classifier was considerably better than with the less populated groups. This was especially clear for the precision of the predictions, which was consistent with the fact that the use of a sufficient number of support vectors helps the classifier learn how to discern disease-associated mutations properly.

**Table 4:** Performance of the classifier when the groups in which UniProt divides the protein kinase superfamily are considered individually

Group	Accuracy (%)	Precision (%)	Recall (%)	MCC
CMGC †	91.5	87.5	8.6	0.1
TKL †	68.7	70.5	70.9	0.4
TK †	71.3	69.7	68.3	0.4
RGC †	58.2	47.9	61.3	0.2
Atypical_PI3-PI4 †	70.6	47.1	100	0.8
STE	96.8	43.7	11.1	0.1
AGC †	90.8	43.3	61.1	0.5
Other	88.9	41.6	95.4	0.9
CK1	97.7	33.3	22.2	0.2
Atypical_Alpha-type	89.9	9.1	88.9	0.8
CAMK †	55.5	8.3	51.9	0.1
Atypical_ADCK	70.0	0	0	0
NEK	100	0	0	0
Atypical_RIO	100	0	0	0
Atypical_PDK-BCKDK	100	0	0	0
Atypical_FAST	100	0	0	0

Performance of the classifier when the groups in which UniProt divides the protein kinase superfamily are considered individually. Groups enriched in disease-associated mutations are indicated by †.

### Analysis of the most relevant features for classification

To evaluate the contribution of each individual feature to the classification, the features were ranked according to the variation in the module of the weight vector of the trained SVM (║ ω ║^2^) when each feature was removed from the set of support vectors. The feature whose removal minimized the variation in module was considered to contribute the least to the hyperplane that separates the two classes of examples (pathogenic/neutral) with a maximum margin. This ranking is shown in Additional file 4 (Supplementary Table 1). The ranking derived from the SVMs has been applied for variable selection in many classification problems [39,40]. According to the SVM-based criteria, the top ranked features are only based on the subset of support vectors that are ‘borderline’ cases.

Gene Ontology (GO) functional annotations contributed the most to the classification. This feature is encoded as the sum of GO terms log-odds ratio, sumGOLOR, to be able to compare between the disease-associated and neutral datasets and corresponds to a classification at the gene-level; it represents the proportion of disease-associated and neutral kinase genes that are annotated with a particular GO term, and it can be used to identify the GO terms characteristic of neutral or disease-prone genes. If the individual terms from the biological process sub-ontology are analyzed, interesting trends can be observed. For example, the most pathogenic biological processes are enriched in terms associated with protein localization, cell proliferation and tissue development, all aspects related to disease and particularly cancers. Pathogenic and neutral genes are differentially enriched in terms from the molecular function sub-ontology. While neutral genes are associated with basic kinase activity functions, disease-associated genes are enriched in terms associated with hormone binding, co-factors and interaction partners. The most representative GO terms for each of the classes are shown in Supplementary Table 2 for neutral genes and Supplementary Table 3 for disease-associated ones (Additional file 4).

The next group of features in the order of relevance for the predictor is linked to the positions that confer specificity at the family level (i.e., the tree-determinants). The calculation of this score is based on our in-house implementation of the S3Det method [35]. However, the current implementation of the method did not provide a continuous measure of tree-determinant characteristics and thus, we implemented this additional possibility. The coincidence of a given residue with the alignment of the rest of the family members, and the differences regarding the sequences outside the subfamily, were measured with an f-score as described in the Methods. Three different scores were calculated: the f-score for the wild type amino acid; the f-score for the mutant residue; and the difference between these two scores as a measure of the relevance of the change introduced.

Following these two important features of the predictor is the Kyte-Doolittle hydrophobicity change, the presence of a PFAM domain (in particular the tyrosine kinase domain), the functional annotation of the residues in SwissProt [33] and PhosphoELM [41], and the evolutionary SIFT score [7] or the amino acid types involved in the change.

Interestingly, among the genome-wide features, some kinase-specific features also emerged as being relevant. For instance, to reinforce the important role of gene-level characterization, classifying kinases into the different groups in KinBase [30] was an important feature (particularly TK, CAMK, CK1 and TKL among the canonical protein kinases, and Alpha-type, ADCK or PI3-PI3 among the atypical ones), as observed previously [19].

### Benchmark of the classifier against other methods

To test the performance of our classifiers, we compared our results with those of five well-established predictors of pathogenicity: SIFT [7], MutationAssessor [9], SNAP [16], SNPs&GO [18] and a kinase-specific method [19]. This set of classifiers represents a wide variety of approaches and scopes: genome-wide and kinase-specific classifiers, different classification approaches (rule-based, neural networks, linear SVMs and radial basis SVMs) and a broad set of classification features. The results of this benchmark are shown in Table 5.

**Table 5:** Summary of the performance of other state-of-the-art classifiers of mutations, either general or kinase-specific

Method	Scope	Accuracy (%)	Precision (%)	Recall (%)	MCC
**KinMut**	Kinase†	83.3	60.0	75.2	0.6
SNPs&GO [18]	Kinase†	82.3	62.8	77.5	0.6
Torkamani [19]	Kinase	77.0	-	-	0.5
MutationAssessor [9]	Kinase†	53.8	41.6	95.6	0.5
SNAP [16]	Kinase†	49.4	34.0	93.1	0.4
SIFT [7]	Kinase†	77.6	37.8	27.9	0.2
SNPs&GO [18]	Genome-wide	82.0	83.0	78.0	0.6
MutationAssessor [9]	Genome-wide	79.0	-	-	-
SNAP [16]	Genome-wide	78.2	76.7	80.2	**-**
SIFT [7]	Genome-wide	68.3	66.1	56.5	0.3

Summary of the performance of other state-of-the-art classifiers of mutations, either general or kinase-specific. Performance was measured in terms of overall accuracy recall and the Matthews correlation coefficient. General methods with which the prediction corresponds to our dataset are marked with †. The remaining results for the classifiers displayed here were taken directly from their original publications

Four genome-wide classifiers SNPs&GO, MutationAssessor, SIFT and SNAP were evaluated with the same kinase dataset as that used to train and evaluate KinMut (Additional file 5). Predictions from SNP&GO and MutationAssessor were obtained through their respective online servers while SIFT and SNAP predictions were retrieved from SNPdbe [42]. Interestingly, when these methods were evaluated with the protein kinase dataset, performance dropped significantly compared to those reported in the original publications for a wider range of protein families (Table 5). It is worth noting that this decrease in the overall performance demonstrates that the protein kinase superfamily is a challenging scenario, justifying the need for kinase-specific classifiers at the cost of scope.

Our predictor generated results with the kinase dataset comparable to those obtained by the best predictor, SNPs&GO. In addition, KinMut performed better than MutationAssessor, SIFT and SNAP (Table 5). Our method yields better results than the kinase-specific method proposed by Torkamani and co-workers, the only method against which a direct comparison can be made. Unfortunately, the original publication did not provide information about recall, precision and the pathogenic mutations resulting from their method. Hence, this method was only compared for accuracy and MCC. Our results are more accurate as we correctly predicted 83.3% of the cases compared to the 77% predicted by the other method. In addition, the correlation coefficient was slightly better in our case, 0.6 compared to 0.5. These results indicate that our choice of features concentrated more predictive power.

### Implementation of the predictor as a web server

We implemented our pipeline to predict mutation pathogenicity in the protein kinase superfamily as a web server, KinMut, which is publicly available at http://kinmut.bioinfo.cnio.es. The server displays the mutations, a summary of the analyzed features and the SVM score for each prediction. Mutations with an SVM score greater than –0.5 are considered damaging, according to the threshold discussed above.

## Discussion

### Performance of the classifier and the benefits of family-specific prediction

It is definitely not easy to compare the capabilities of different prediction methods and many technical difficulties arise [6]. Choosing an objective testing dataset is the most difficult, especially when the datasets used in the original publications are not equivalent. Moreover, increased predictive capabilities would be expected if the testing dataset had already been presented to the classifier during the learning process. This is very likely the case for the kinase dataset, which is a strict subset of the most commonly used training dataset [36]. Consequently, the results presented represent a means to understand the capabilities of KinMut in its context rather than a detailed ranking of prediction methods.

KinMut achieves a level of performance similar to that obtained by the best predictors, SNPs&GO [18] and it outperforms other reference methods such as SIFT [7], SNAP [16] and MutationAssessor [9] when evaluated within the framework of the kinase dataset. It also achieves better results than Torkamani’s kinase-specific [19] method.

Interestingly, we achieved results comparable to the best classifier, SNPs&GO, whose capability we probably overestimated since it has been trained with all the mutations in UniProt, which are very likely included in the subset of kinase mutations, what gives SNPs&GO some advantage since the classifier had already been presented with the mutations. Thus, given that it is not possible to train the predictor without the kinase mutations, we assume this artifactual increase in performance to be acceptable for the analysis presented here.

Probably, the similarity in the performance is given by the use of GO terms since both methods - even though they differ in their scope and implementation - benefit from functional information encoded as GO terms at the gene level, which is the most discriminative feature of our classifier.

Our predictor performs beyond the capabilities of the only method against which an utterly fair comparison can be conducted, Torkamani’s kinase-specific predictor [43], at least in terms of accuracy and correlation. Unfortunately, the authors of this method did not provide information about its recall, precision and output, to enable a better comparison to have been made. Interestingly, Torkamani’s and our classifiers share several properties: amino acid types; kinase group membership, which the authors state to be critical for classification; biochemical properties such as the Kyte-Doolittle hydrophobicity index; and evolutionary conservation. In spite of these similarities, Torkamani’s method does not benefit from intra-family specificity positions or from GO annotations, which we have shown to be crucial for prediction (see above). This might have caused the differences in performance observed.

Moreover, it is not surprising that KinMut predicts more accurately than SIFT, being the latter used as a classification feature. The difference in performance should be attributed to the predictive power of the additional features and the machine-learning approach.

Current genome-wide predictors of mutation pathogenicity perform well on average, probably because they can use the huge amount of mutation data available. However, most of these predictors only exploit the subset of features that could be generalized to the entire range of protein families in the human proteome, which constitutes an intrinsic limitation. By contrast, family-specific predictors, such as the method presented here for the protein kinase superfamily, can overcome this limitation and benefit from features that apply only to the protein family of interest. These family-specific features might capture aspects of pathogenicity that are unique to that given protein family. We explored the basis of using kinase-specific features, such as kinase group membership, annotation with certain GO terms and the presence of determined PFAM domains, which are relevant for predicting pathogenicity in the protein kinase superfamily. Accordingly, the performance of genome-wide methods decreases when they are confronted with the set of kinase mutations.

Indeed, the family-specific nature of our method allowed us to explore features that are unique to the protein kinase superfamily, retaining valuable information on mutation pathogenicity. In our case, membership to a particular kinase group and the occurrence of mutations in the catalytic protein kinase domain were important features that are unique to the protein kinase superfamily. This is in full agreement with previous observations that reached similar conclusions [19,43].

The results provided here reinforce the idea that for well-studied families like the kinase superfamily, family-specific classifiers can use unique features that are only valid in the context of this specific superfamily, thereby improving performance over general purpose methods.

Regarding the dissimilarities between the different branches of the kinase phylogenetic tree, we demonstrated that more accurate results were obtained for groups with sufficient data that allowed the classifier to learn to weight the importance of the individual contribution of the features precisely. Moreover, this group membership was one of the most triggering features of our classification. There are groups in which very few (or even no) pathogenic mutations have been described and as such, in these cases group membership is a powerful means to predict neutral mutations. However, since the mutational landscape is far from complete, we cannot discern whether this is a reliable scenario (where these kinase groups do not elicit pathogenicity) or rather an artifact due to a gap in our current knowledge that will be filled when new mutations are discovered. Indeed, the uneven, heterogenic, distribution of experimental evidence regarding the different kinase groups does not only affect the number of mutations discovered but also, the quality and thoroughness of features such as GO or UniProt annotations, which is very likely to influence the predictive capacity of our system. We expect that, in the near future, ongoing genomic projects will help us understand the links between mutations in all the kinase groups and disease, thereby boosting the capability of kinase-specific prediction methods beyond the limits of current highly populated groups.

The current practical use of our method is as a component of a system that ranks mutations by their potential importance in the context of cancer genome analysis in a preclinical environment.

## Methods

### Mutation dataset

The mutation data used here was derived directly from UniProt [33] (release 2011_01; Jan 11, 2011) after applying the following constraints:

1. The protein is annotated as a protein kinase in UniProt.

2. It is a human protein.

3. The mutation corresponds to non-synonymous, non-truncating single point coding mutations. Other mutation types such as insertions, deletions, copy number alterations, truncating and silent mutations were not considered in this analysis.

The use of a UniProt derived dataset has recently been benchmarked for a number of classifiers with satisfactory results [36].

Following this pre-filtering step, we classified the mutations as disease or neutral mutations according to the annotation in UniProt. There is a third group in UniProt that aggregates the mutations for which insufficient information is available, mutations that were ruled out of this analysis. After the whole selection process, the ‘disease dataset’ that includes mutations for which there is experimental evidence of their disease association, contained 865 mutations in 65 human kinases (Additional file 6). By contrast, the ‘neutral dataset’ that contains mutations with no experimental proof of association to disease, contained 2,627 mutations in 447 human protein kinases (Additional file 7). For classification purposes, 142 sequence features describe each mutation.

### Implementation and optimization of the classifier

To implement the Support Vector Machine classifier we used the SVMLight (http://www.cs.Cornell.edu/people/tj/svm_light/) package with a radial basic function (RBF) kernel:

*K*(*x_i_*, *x_j_*) = *exp*(–*G*║*x_i_* – *x_j_*║^2^))

In this manner, two parameters are crucial to the performance of the classifier: the soft-margin penalty (C) and the radius (γ). These parameters were optimized using a grid search where an exhaustive evaluation was carried out for values ranging between 0 ≤ *C* ≤ 8 in 1 unit steps, and 10^–4^ ≤ γ ≤ 10^–2^ increasing by 5 · 10^–4^ after each run. We used the f-score as optimization criteria. In order to ensure fairness, we conducted a k-fold cross-validation analysis for each of the C-gamma tuples. We randomly distributed the mutations in 10 different subsets: 8 sets were used for training, one set for validation and 1 set for evaluation of the performance. We forced each of the subsets as evaluation set (which was kept apart at this stage), and we rotated the other 9 so that each of them could be used as validation set while the remaining 8 constitute the training set. Each independent run provides a partial f-score, and the mean across the 90 partial f-scores provides the average f-score of that given C-gamma tuple that was used as selection criteria. This approach ensures that the f-score is maximized and there are no biases in the selection of the datasets, while we avoid over-fitting by evaluating the classifier with mutations that had already been presented to it during the training process.

### Evaluation of performance

The performance of our classifier was evaluated using a 10-fold cross-validation approach as described above. The process is repeated 90 times to ensure that all subsets of mutations are used for each purpose. The classifier’s performance is averaged across all combinations in order to avoid over-interpreting the quality of the method. The efficiency of the classifier can be assessed in many ways and here we describe the most illustrative ones.

Hereafter, we will refer to the following abbreviations:

**TP** True positives, correctly predicted disease-associated mutations.

**FP** False positives, neutral mutations predicted as disease prone.

**TN** True negatives, correctly predicted neutral mutations.

**FN** False negatives, disease-associated mutations predicted as neutral.

Accuracy accounts for the fraction of mutations correctly predicted in function of the total number of mutations.

Recall, also referred to as sensitivity by other authors, accounts for the proportion of correctly predicted disease-associated mutations in function of all the disease-associated mutations in the dataset.

Precision accounts for the proportion of correctly predicted disease-associated mutations with respect to all the predicted disease-associated mutations.

The F-score is a measure of the accuracy of the classification. It considers both the precision and the recall in a single representative score for evaluation purposes.

The Matthews Correlation Coefficient (MCC) was calculated according to the following formula:

### Classification feature: membership to a kinase group

In order to cluster the kinases according to the groups they belong to, two different classification schemes were used. The KinBase resource [30] constitutes the currently accepted classification scheme of eukaryotic protein kinases. According to KinBase, kinases are categorized as ‘conventional’ protein kinases (ePKs) or ‘atypical’ protein kinases (aPKs). The ePKs form the largest group and they have been subdivided into 8 groups according to sequence similarity, the presence of accessory domains and by considering different modes of regulation. The eight ePK groups defined in KinBase correspond to: the AGC group (including cyclic-nucleotide and calcium-phospholipid-dependent kinases, ribosomal S6-phosphorylating kinases, G protein-coupled kinases and close relatives of these kinases); the CAMKs (calmodulin-regulated kinases); the CK1 group (casein kinase 1 and close relatives); the CMGC group (including cyclin-dependent kinases, mitogen-activated protein kinases, CDK-like kinases and glycogen synthase kinase); the RGC group (receptor guanylate cyclase kinases); the STE group (MAPK cascade kinases); the TK (tyrosine kinase) and the TKL (TK-like), which are a group of serine-threonine kinases resembling TKs. Another broad miscellaneous group, called ‘Other’, is also considered for those proteins that do not fit in any of these predefined categories. By contrast, UniProt [33] provides a classification scheme that includes the same groups included in KinBase along with the additional groups, NEK and STG, making a total of 11 groups. The vector of features submitted to the classifier contains a position for each of the groups in the latter scheme. The values are encoded as 1 for the group to which the kinase housing the mutation belongs to, and 0 for the rest. A similar approach was followed by Torkamani and Schork [19].

### Classification feature: Gene Ontology log-odds ratio

The Gene Ontology log-odds ratio (GOLOR) was used to classify the mutations as pathogenic (disease-associated) or neutral according to the annotations regarding the function of the genes in which they exist. To compute the score, we retrieved all the terms associated to the kinases in our dataset from the 3 sub-ontologies in Gene Ontology [31] (Molecular Function, Biological Process, Cell Component). The ontologies were followed towards the root of each ontology in order to include all parental terms in the calculation. Note that ‘part-of’ relationships were discarded and only ‘is-a’ links were considered. For each of the kinase genes, the sum of the Gene Ontology Log Odds Ratio (sumGOLOR) was computed as follows:

Where disease-associated kinase genes are those with at least one reported disease-associated mutation and neutral kinase genes are those with no reported disease-associated mutation. In order to resolve undetermined ratios, frequencies equal to 0 were artificially set to 10^–9^. A similar approach with slight changes in the algorithm is followed in two other methods: CanPredict [13,44] and SNPs&GO [18].

### Classification feature: PFAM domains

The position of the different domains in the sequence of the human protein kinome was extracted from the swisspfam file in PFAM [32]. A binary position in the vector was created for each of the 117 different domains in the protein kinase family, where 1 means that the mutation is in a position that was characterized as part of that domain, and otherwise it is attributed a value 0. An additional binary position in the vector, PFAM_any, was created to record whether the position belongs to at least one PFAM domain. This is a simplified version of the implementation by other authors [13,16,19].

### Classification feature: amino acid type and change in hydrophobicity

Each amino acid type was encoded at 20 positions in the vector, where the wild-type residue is encoded as 1 and its mutant counterpart is encoded as -1.

The rest of values remain as 0 for classification purposes. An additional position was encoded to represent the change in the Kyte-Doolittle hydrophobicity index [45].

### Classification feature: UniProt annotation

UniProt [33] provides a detailed description of the residues for a number of proteins in the database. We considered 5 different classes of residue annotation to be relevant:

1. Catalytic site (including residues annotated as SITE, BINDING, ACT_SITE, METAL and NP_BIND: refer to the UniProt help pages for a detailed description of the annotations)

2. Disulfide bond (DISULFID)

3. Post-Translational Modifications (MOD_RES, SIGNAL)

4. Residues with special interest (MUTAGEN)

5. Transmembrane regions (TRANSMEM).

A binary input corresponding to each of these categories was added to the classification vector. In addition, two additional positions were added: one that corresponds to a positive match in at least one category from the catalytic site class, while the other corresponds to a positive match in at least one of the categories described above. A similar approach was followed previously [10,14,16,38].

### Classification feature: phosphorylation sites

PhosphoELM [41] is a database of eukaryotic phosphorylation sites. This resource includes manually curated information derived from the literature, as well as high-throughput analyses for 1,232 phosphorylation sites in 287 human kinases present in our dataset. Since out of the 20 potential residues only 3 can be phosphorylated (Ser, Tyr, Thr), this feature was encoded for the classifier according to 3 different states: 1 represents a reported residue amenable for phosphorylation, 0 if the residue is a Ser, Thr or Tyr that is not phosphorylated, and -1 for the remaining residues.

### Classification feature: catalytic sites

FireDB [34] is a database of known functionally relevant residues. It includes both biologically relevant data filtered from the close atomic contacts in 3D crystal structures and manually annotated catalytic residues. The presence of a mutation in the catalytic site of a protein was encoded in the classifier as a binary input, whereby 1 means that the mutation is part of the catalytic site, 0 otherwise.

### Classification feature: evolutionary information

In order to capture the similarity between closely related proteins and thereby identify potentially deleterious changes, we included the SIFT score in our feature vector [7]. This method relies on the normalized probabilities for all possible residue substitutions at each position of a multiple sequence alignment of homologous proteins. The score can be easily translated into a binary output where values <0.05 are considered deleterious. Consequently, both the binary and the continuous versions of the score were computed in order to provide more discriminating results. Additionally, the number of sequences in the alignment at the position of interest was also considered. Since it was introduced in 2001, this method has been successfully incorporated in several predictors of pathogenicity [13,14,16,19].

### Classification feature: specificity determining positions

Those positions occupied by conserved residues within groups of proteins in a family sharing a common general specificity that differs between groups can be used as a proxy for the regions accounting for subfamily specificity. SDPs, also referred as tree-determinants on occasion, were calculated using a simplified version of the in-house S3Det predictor [35]. In our implementation, the f-score associated to the wild-type and mutant residues in the classification of the subfamilies calculated from the sequences in the PFAM alignments was encoded in the classification vector. An additional third position represented the difference between these two scores. This difference represents the change (increase or decrease) in agreement with the subfamily introduced by the mutation.

## Conclusions

Our choice of features and datasets makes the method especially relevant in the context of kinase mutations and their intrinsic role in cancer biology. In our particular case, the membership to a particular kinase group or the occurrence of the mutations at the catalytic protein kinase domain arise as important features that are unique to the protein kinase superfamily. This is in full agreement with previous observations [19]. The family-specific character of the KinMut classifier allowed us to introduce features that are unique to the protein family of interest and that retain valuable information about the pathogenicity of the mutation.

## Competing interests

The authors declare that they have no competing interests.

## Author’s contributions

AV and JMGI designed the experiment. AP implemented the classifier. JMGI and AP trained and evaluated the classifier. JMGI benchmarked the classifier against the other methods. MV and JMGI designed and developed the web server. JMGI and AV wrote the paper. All the authors read and approved this manuscript.

## Supplementary Material

Additional file 1 **Supplementary figure 1.png** Grid optimization of the predictive power of the classifier (all groups): F-score. We exhaustively tested the two most critical parameters of the SVM’s radial basis kernel: soft-margin (*C*) and radius (γ). The average f-score across the entire set of k-folds was chosen as a scoring function for the optimization. The optimal values used for the analyses were *C* = 3 and γ = 6 · 10^–4^ when all groups in the kinase superfamily were considered. Click here for file

Additional file 2 **Supplementary figure 2.png** Grid optimization of the predictive power of the classifier (all groups): AUC. We exhaustively tested the two most critical parameters of the SVM’s radial basis kernel: soft-margin (*C*) and radius (γ). The average area under the curve (AUC) across the entire set of k-folds was chosen as a scoring function for the optimization. The optimal values correspond to *C* = 2 and γ = 6 · 10^–4^. Click here for file

Additional file 3 **Supplementary figure 3.png** Grid optimization of the predictive power of the classifier (populated groups): F-score. Grid optimization of the predictive power of the classifier when only the groups with a reasonable number of reported disease-associated mutations are considered. We exhaustively tested soft-margin (*C*) and γ. The average f-score across the entire set of k-folds was chosen as the scoring function for the optimization. The optimal values used during the analyses were *C* = 8 and γ = 10^–4^. Click here for file

Additional file 4 **Supplementary tables.pdf** Supplementary Table 1: Ranking of the features according to their contribution to classification. Supplementary Table 2: Most representative GO terms to classify kinase genes as neutral. Supplementary Table 3: Most representative GO terms to classify kinase genes as disease-associated. Click here for file

Additional file 5 **Supplementary figure 5.png** Benchmark of the classifiers with a common kinase dataset. Evaluation of the prediction capabilities of the four genome-wide classifiers (SNPs&GO, MutationAssessor, SIFT and SNAP) in comparison to KinMut. All predictors were evaluated with the same kinase dataset. Predictions from SNP&GO and MutationAssessor were obtained through their respective online servers while SIFT and SNAP predictions were retrieved from SNPdbe [42]. The dashed line represents the theoretical random predictor. Click here for file

Additional file 6 **Variants.disease.txt** Dataset of disease-associated mutations used to train and to evaluate the predictor. Click here for file

Additional file 7 **Variants.neutral.txt** Dataset of neutral mutations used in to train and to evaluate the predictor. Click here for file

## References

[B1] Sjöblom T Jones S Wood LD Parsons DW Lin J Barber TD Mandelker D Leary RJ Ptak J Silliman N Szabo S Buckhaults P Farrell C Meeh P Markowitz SD Willis J Dawson D Willson JKV Gazdar AF Hartigan J Wu L Liu C Parmigiani G Park BH Bachman KE Papadopoulos N Vogelstein B Kinzler KW Velculescu VE The consensus coding sequences of human breast and colorectal cancers Science 2006 314 5797 268 74 10.1126/science.1133427 16959974

[B2] Wood LD Parsons DW Jones S Lin J Sjöblom T Leary RJ Shen D Boca SM Barber TD Ptak J Silliman N Szabo S Dezso Z Ustyanksky V Nikolskaya T Nikolsky Y Karchin R Wilson PA Kaminker JS Zhang Z Croshaw R Willis J Dawson D Shipitsin M Willson JKV Sukumar S Polyak K Park BH Pethiyagoda CL Pant PVK Ballinger DG Sparks AB Hartigan J Smith DR Suh E Papadopoulos N Buckhaults P Markowitz SD Parmigiani G Kinzler KW Velculescu VE Vogelstein B The genomic landscapes of human breast and colorectal cancers Science 2007 318 5853 1108 13 10.1126/science.1145720 17932254

[B3] Greenman C Stephens P Smith R Dalgliesh GL Hunter C Bignell G Davies H Teague J Butler A Stevens C Edkins S O’Meara S Vastrik I Schmidt EE Avis T Barthorpe S Bhamra G Buck G Choudhury B Clements J Cole J Dicks E Forbes S Gray K Halliday K Harrison R Hills K Hinton J Jenkinson A Jones D Menzies A Mironenko T Perry J Raine K Richardson D Shepherd R Small A Tofts C Varian J Webb T West S Widaa S Yates A Cahill DP Louis DN Goldstraw P Nicholson AG Brasseur F Looijenga L Weber BL Chiew YE DeFazio A Greaves MF Green AR Campbell P Birney E Easton DF Chenevix-Trench G Tan MH Khoo SK Teh BT Yuen ST Leung SY Wooster R Futreal PA Stratton MR Patterns of somatic mutation in human cancer genomes Nature 2007 446 7132 153 8 10.1038/nature05610 17344846 PMC2712719

[B4] Baudot A Real F Izarzugaza J Valencia A From cancer genomes to cancer models: bridging the gaps EMBO Rep 2009 10.1038/embor.2009.46 PMC2672900 19305388

[B5] Karchin R Next generation tools for the annotation of human SNPs Brief Bioinformatics 2009 10 35 52 19181721 10.1093/bib/bbn047 PMC2638621

[B6] Cline M Karchin R Using bioinformatics to predict the functional impact of SNVs Bioinformatics 2010 10.1093/bioinformatics/btq695 PMC3105482 21159622

[B7] Ng PC Henikoff S Predicting deleterious amino acid substitutions Genome Res 2001 11 5 863 74 10.1101/gr.176601 11337480 PMC311071

[B8] Clifford RJ Edmonson MN Nguyen C Buetow KH Large-scale analysis of non-synonymous coding region single nucleotide polymorphisms Bioinformatics 2004 20 7 1006 14 10.1093/bioinformatics/bth029 14751981

[B9] Reva B Antipin Y Sander C Predicting the functional impact of protein mutations: application to cancer genomics Nucleic Acids Res 2011 10.1093/nar/gkr407 PMC3177186 21727090

[B10] Ramensky V Bork P Sunyaev S Human non-synonymous SNPs: server and survey Nucleic Acids Res 2002 30 17 3894 900 10.1093/nar/gkf493 12202775 PMC137415

[B11] Wang Z Moult J SNPs, protein structure, and disease Hum Mutat 2001 17 4 263 70 10.1002/humu.22 11295823

[B12] Krishnan VG Westhead DR A comparative study of machine-learning methods to predict the effects of single nucleotide polymorphisms on protein function Bioinformatics 2003 19 17 2199 209 10.1093/bioinformatics/btg297 14630648

[B13] Kaminker JS Zhang Y Waugh A Haverty PM Peters B Sebisanovic D Stinson J Forrest WF Bazan JF Seshagiri S Zhang Z Distinguishing cancer-associated missense mutations from common polymorphisms Cancer Res 2007 67 2 465 73 10.1158/0008-5472.CAN-06-1736 17234753

[B14] Wainreb G Ashkenazy H Bromberg Y Starovolsky-Shitrit A Haliloglu T Ruppin E Avraham KB Rost B Ben-Tal N MuD: an interactive web server for the prediction of non-neutral substitutions using protein structural data Nucleic Acids Res 2010 38 Suppl W523 8 20542913 10.1093/nar/gkq528 PMC2896130

[B15] Ferrer-Costa C Orozco M de la Cruz X Characterization of disease-associated single amino acid polymorphisms in terms of sequence and structure properties J Mol Biol 2002 315 4 771 86 10.1006/jmbi.2001.5255 11812146

[B16] Bromberg Y Rost B SNAP: predict effect of non-synonymous polymorphisms on function Nucleic Acids Res 2007 35 11 3823 35 10.1093/nar/gkm238 17526529 PMC1920242

[B17] Adzhubei IA Schmidt S Peshkin L Ramensky VE Gerasimova A Bork P Kondrashov AS Sunyaev SR A method and server for predicting damaging missense mutations Nat Methods 2010 7 4 248 9 10.1038/nmeth0410-248 20354512 PMC2855889

[B18] Calabrese R Capriotti E Fariselli P Martelli PL Casadio R Functional annotations improve the predictive score of human disease-related mutations in proteins Hum Mutat 2009 30 8 1237 44 10.1002/humu.21047 19514061

[B19] Torkamani A Schork NJ Accurate prediction of deleterious protein kinase polymorphisms Bioinformatics 2007 23 21 2918 25 10.1093/bioinformatics/btm437 17855419

[B20] Yue P Li Z Moult J Loss of protein structure stability as a major causative factor in monogenic disease J Mol Biol 2005 353 2 459 73 10.1016/j.jmb.2005.08.020 16169011

[B21] Karchin R Diekhans M Kelly L Thomas DJ Pieper U Eswar N Haussler D Sali A LS-SNP: large-scale annotation of coding non-synonymous SNPs based on multiple information sources Bioinformatics 2005 21 12 2814 20 10.1093/bioinformatics/bti442 15827081

[B22] González-Pérez A López-Bigas N Improving the assessment of the outcome of nonsynonymous SNVs with a consensus deleteriousness score, Condel Am J Hum Genet 2011 88 4 440 9 10.1016/j.ajhg.2011.03.004 21457909 PMC3071923

[B23] Izarzugaza JMG Krallinger M Rodriguez-Penagos C Valencia A Extraction of human kinase mutations from literature, databases and genotyping studies BMC Bioinformatics 2009 10 Suppl 8 S1 10.1186/1471-2105-10-S8-S1 19758464 PMC2745582

[B24] Hurst J McMillan L Porter C Allen J Fakorede A Martin A The SAAPdb web resource: A large-scale structural analysis of mutant proteins Hum Mutat 2009 10.1002/humu.20898 19191322

[B25] Lahiry P Torkamani A Schork NJ Hegele RA Kinase mutations in human disease: interpreting genotype-phenotype relationships Nat Rev Genet 2010 11 60 74 10.1038/nrg2707 20019687

[B26] Greenman C Wooster R Futreal PA Stratton MR Easton DF Statistical analysis of pathogenicity of somatic mutations in cancer Genetics 2006 173 4 2187 98 10.1534/genetics.105.044677 16783027 PMC1569711

[B27] Stratton MR Campbell PJ Futreal PA The cancer genome Nature 2009 458 7239 719 24 10.1038/nature07943 19360079 PMC2821689

[B28] Izarzugaza JMG McMillan LEM Baresic A Orengo CA Martin ACR Valencia A Characterization of pathogenic germline mutations in human Protein Kinases BMC Bioinformatics 2011 12 Suppl 4 10.1186/1471-2105-12-S4-S1 PMC3194193 21992016

[B29] Izarzugaza J Redfern O Orengo C Valencia A Cancer-associated mutations are preferentially distributed in protein kinase functional sites Proteins 2009 10.1002/prot.22512 19626714

[B30] Manning G Whyte DB Martinez R Hunter T Sudarsanam S The protein kinase complement of the human genome Science 2002 298 5600 1912 34 10.1126/science.1075762 12471243

[B31] Ashburner M Ball CA Blake JA Botstein D Butler H Cherry JM Davis AP Dolinski K Dwight SS Eppig JT Harris MA Hill DP Issel-Tarver L Kasarskis A Lewis S Matese JC Richardson JE Ringwald M Rubin GM Sherlock G Gene ontology: tool for the unification of biology. The Gene Ontology Consortium Nat Genet 2000 25 25 9 10.1038/75556 10802651 PMC3037419

[B32] Finn RD Mistry J Tate J Coggill P Heger A Pollington JE Gavin OL Gunasekaran P Ceric G Forslund K Holm L Sonnhammer ELL Eddy SR Bateman A The Pfam protein families database Nucleic Acids Res 2010 38 Database issue D211 22 19920124 10.1093/nar/gkp985 PMC2808889

[B33] Bairoch A Boeckmann B Ferro S Gasteiger E SwissProt: juggling between evolution and stability Brief Bioinformatics 2004 5 39 55 10.1093/bib/5.1.39 15153305

[B34] López G Valencia A Tress ML FireDB–a database of functionally important residues from proteins of known structure Nucleic Acids Res 2007 35 Database issue D219 23 17132832 10.1093/nar/gkl897 PMC1716728

[B35] Rausell A Juan D Pazos F Valencia A Protein interactions and ligand binding: from protein subfamilies to functional specificity Proc Natl Acad Sci USA 2010 107 5 1995 2000 10.1073/pnas.0908044107 20133844 PMC2808218

[B36] Care MA Needham CJ Bulpitt AJ Westhead DR Deleterious SNP prediction: be mindful of your training data! Bioinformatics 2007 23 6 664 72 10.1093/bioinformatics/btl649 17234639

[B37] Yue P Melamud E Moult J SNPs3D: candidate gene and SNP selection for association studies BMC Bioinformatics 2006 7 166 10.1186/1471-2105-7-166 16551372 PMC1435944

[B38] Ye ZQ Zhao SQ Gao G Liu XQ Langlois RE Lu H Wei L Finding new structural and sequence attributes to predict possible disease association of single amino acid polymorphism (SAP) Bioinformatics 2007 23 12 1444 50 10.1093/bioinformatics/btm119 17384424

[B39] Guyon I Weston J Barnhill S Vapnik V Gene Selection for Cancer Classification using Support Vector Machines Support Vector Machines 2002 46 389 422

[B40] Rakotomamonjy A Variable selection using SVM-based criteria JMLR 2003 3 1357 70

[B41] Diella F Gould CM Chica C Via A Gibson TJ Phospho.ELM: a database of phosphorylation sites-update 2008 Nucleic Acids Res 2008 36 Database issue D240 4 17962309 10.1093/nar/gkm772 PMC2238828

[B42] Schaefer C Meier A Rost B Bromberg Y SNPdbe: constructing an nsSNP functional impacts database Bioinformatics 2012 28 4 601 2 10.1093/bioinformatics/btr705 22210871 PMC3278761

[B43] Torkamani A Kannan N Taylor SS Schork NJ Congenital disease SNPs target lineage specific structural elements in protein kinases Proc Natl Acad Sci USA 2008 105 26 9011 6 10.1073/pnas.0802403105 18579784 PMC2449356

[B44] Kaminker JS Zhang Y Watanabe C Zhang Z CanPredict: a computational tool for predicting cancer-associated missense mutations Nucleic Acids Res 2007 35 Web Server issue W595 8 17537827 10.1093/nar/gkm405 PMC1933186

[B45] Kyte J Doolittle RF A simple method for displaying the hydropathic character of a protein J Mol Biol 1982 157 105 32 10.1016/0022-2836(82)90515-0 7108955

