# Supplementary figures and images for: Prioritization of pathogenic mutations in the protein kinase superfamily
Source: BMC Genomics. 2012 Jun 18;13(Suppl 4):S3. doi: 10.1186/1471-2164-13-S4-S3 (PMC3303724; doi:10.1186/1471-2164-13-S4-S3)

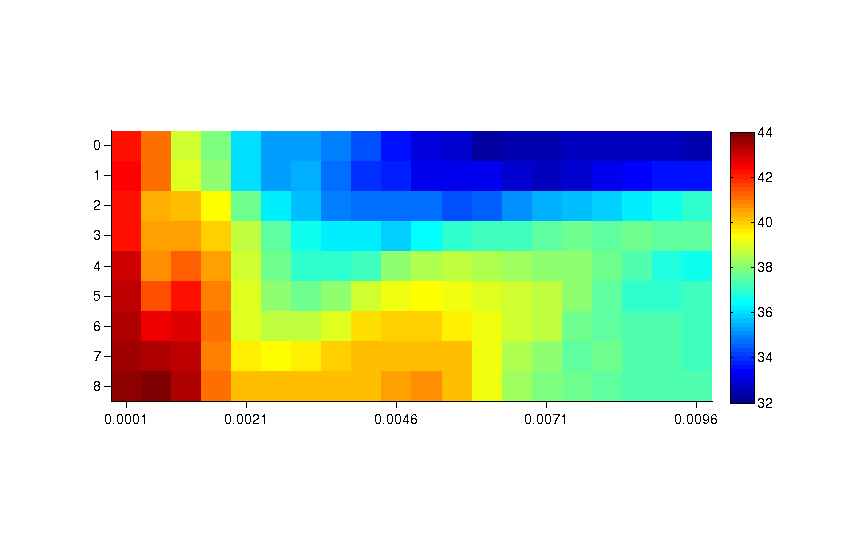

Supplement: Additional file 1 — Supplementary figure 1.png Grid optimization of the predictive power of the classifier (all groups): F-score. We exhaustively tested the two most critical parameters of the SVM’s radial basis kernel: soft-margin (C) and radius (γ). The average f-score across the entire set of k-folds was chosen as a scoring function for the optimization. The optimal values used for the analyses were C = 3 and γ = 6 · 10–4 when all groups in the kinase superfamily were considered. [file 1471-2164-13-S4-S3-S1.png]

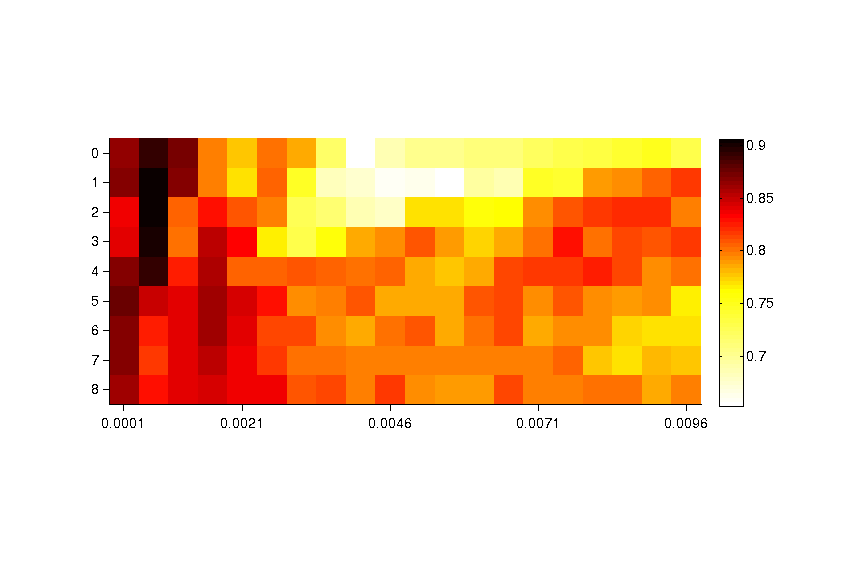

Supplement: Additional file 2 — Supplementary figure 2.png Grid optimization of the predictive power of the classifier (all groups): AUC. We exhaustively tested the two most critical parameters of the SVM’s radial basis kernel: soft-margin (C) and radius (γ). The average area under the curve (AUC) across the entire set of k-folds was chosen as a scoring function for the optimization. The optimal values correspond to C = 2 and γ = 6 · 10–4. [file 1471-2164-13-S4-S3-S2.png]

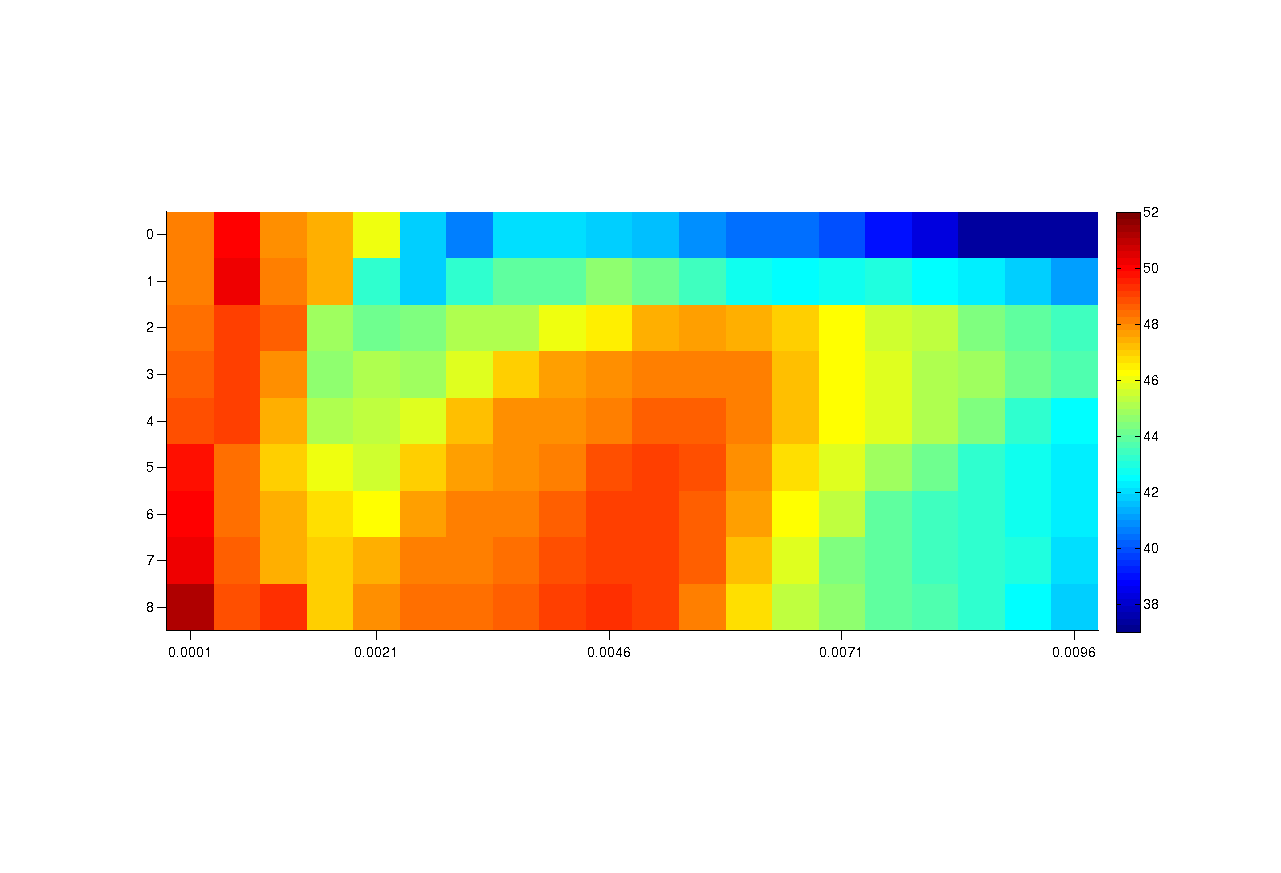

Supplement: Additional file 3 — Supplementary figure 3.png Grid optimization of the predictive power of the classifier (populated groups): F-score. Grid optimization of the predictive power of the classifier when only the groups with a reasonable number of reported disease-associated mutations are considered. We exhaustively tested soft-margin (C) and γ. The average f-score across the entire set of k-folds was chosen as the scoring function for the optimization. The optimal values used during the analyses were C = 8 and γ = 10–4. [file 1471-2164-13-S4-S3-S3.png]

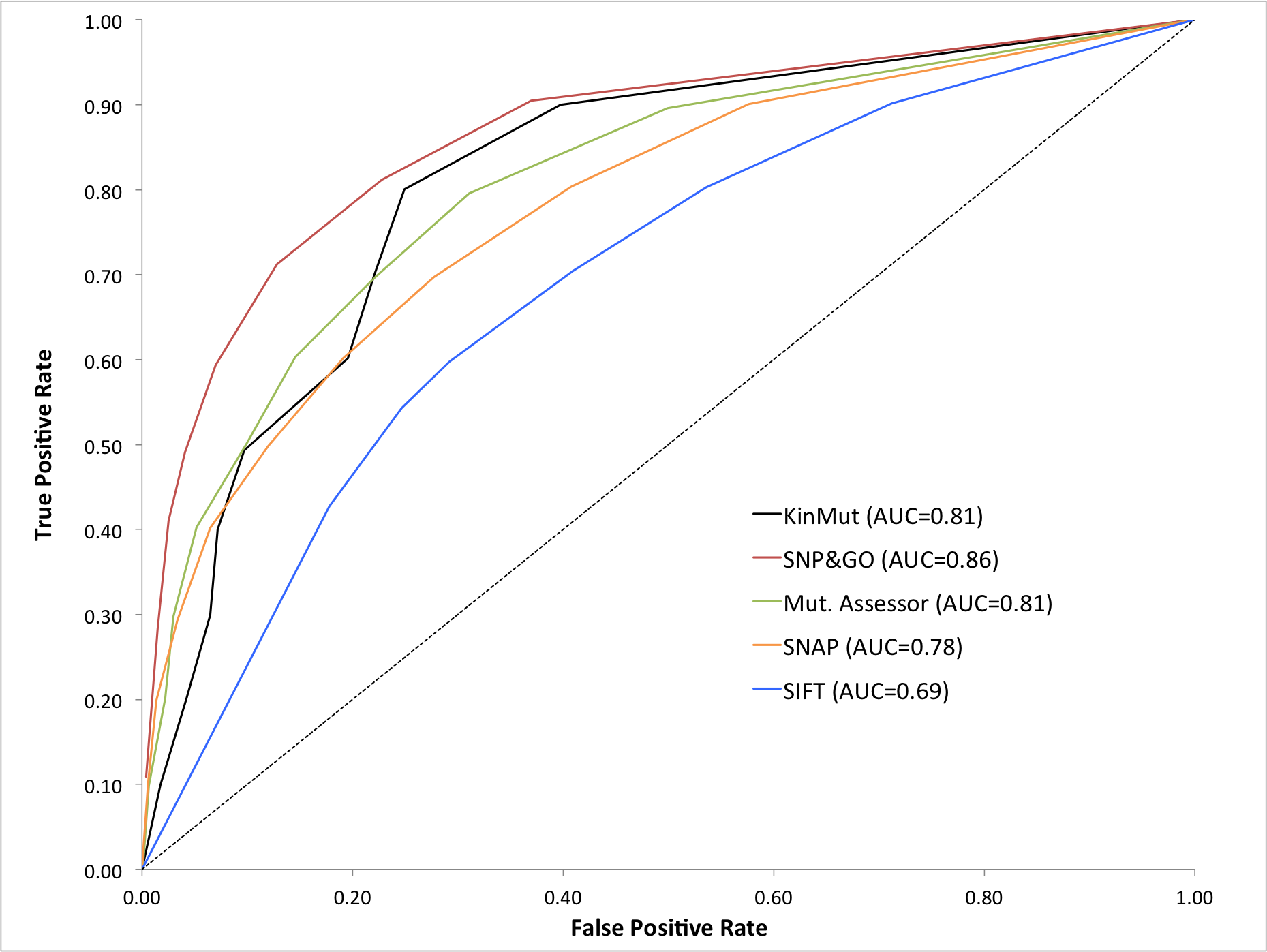

Supplement: Additional file 5 — Supplementary figure 5.png Benchmark of the classifiers with a common kinase dataset. Evaluation of the prediction capabilities of the four genome-wide classifiers (SNPs&GO, MutationAssessor, SIFT and SNAP) in comparison to KinMut. All predictors were evaluated with the same kinase dataset. Predictions from SNP&GO and MutationAssessor were obtained through their respective online servers while SIFT and SNAP predictions were retrieved from SNPdbe [42]. The dashed line represents the theoretical random predictor. [file 1471-2164-13-S4-S3-S5.png]
