# Supplementary material for: Prioritization of pathogenic mutations in the protein kinase superfamily
Source: BMC Genomics. 2012 Jun 18;13(Suppl 4):S3. doi: 10.1186/1471-2164-13-S4-S3 (PMC3303724; doi:10.1186/1471-2164-13-S4-S3)
Supplement: Additional file 4 — Supplementary tables.pdf Supplementary Table 1: Ranking of the features according to their contribution to classification. Supplementary Table 2: Most representative GO terms to classify kinase genes as neutral. Supplementary Table 3: Most representative GO terms to classify kinase genes as disease-associated. [file 1471-2164-13-S4-S3-S4.pdf]

Table 1: Ranking of the features according to their contribution to the classification.

Rank	Feature Description	Rank	Feature Description
1	sumGOLOR	21	pfam_PF07714 (PKinase_tyr)
2	Tree Determinants (difference)	22	PhosphoELM
3	Tree Determinants (wild-type)	23	kinasegroup _CK1
4	Kyte Doolittle hydrophobicity	24	SWannotation: Disulphide bond
5	Tree Determinants (mutant)	25	SIFTscore
6	Leucine	26	Glutamate
7	pfam_any	27	Tryptophan
8	SIFTscore (binned)	28	Phenylalanine
9	Alanine	29	pfam_PF00041 (Fibronectin)
10	kinasegroup _TK	30	kinasegroup _TKL
11	Valine	31	kinasegroup _Atypical_Alpha-type
12	Arginine	32	Glutamine
13	Serine	33	FireDB
14	Lysine	34	Aspartate
15	SWannoation: Any	35	kinasegroup _Atypical_ADCK
16	Histidine	36	Isoleucine
17	Asparagine	37	pfam_PF00023 (Ank)
18	Methionine	38	pfam_PF01403 (Sema)
19	Kinasegroup_CAMK	39	kinasegroup _Atypical_PI3-PI4
20	Threonine	40	Glycine

Table 2: Most representative GO terms to classify kinase genes as neutral.

#GO	%neu	sumGOLOR	Description
GO:0019901	4.62	-32.1	(MF) protein kinase binding
GO:0046328	4.62	-32.1	(BP) regulation of JNK cascade
GO:0070302	4.62	-32.1	(BP) regulation of stress-activated protein kinase signaling cascade
GO:0005083	3.85	-31.84	(MF) small GTPase regulator activity
GO:0043506	3.85	-31.84	(BP) regulation of JUN kinase activity
GO:0008134	3.59	-31.74	(MF) transcription factor binding
GO:0043507	3.59	-31.74	(BP) positive regulation of JUN kinase activity
GO:0005794	3.08	-31.52	(CC) Golgi apparatus
GO:0007257	3.08	-31.52	(BP) activation of JUN kinase activity
GO:0051098	3.08	-31.52	(BP) regulation of binding
GO:0030528	2.56	-31.26	(MF) transcription regulator activity
GO:0051090	2.56	-31.26	(BP) regulation of transcription factor activity
GO:0051101	2.56	-31.26	(BP) regulation of DNA binding
GO:0090046	2.56	-31.26	(BP) regulation of transcription regulator activity
GO:0004707	2.31	-31.1	(MF) MAP kinase activity
GO:0019207	2.31	-31.1	(MF) kinase regulator activity
GO:0019887	2.31	-31.1	(MF) protein kinase regulator activity

%neu: Percentage of neutral genes that are annotated with a given GO term. MF: Molecular Function, BP: Biological Process, CC: Cellular Component.

Table 3: Most representative GO terms to classify kinase genes as disease-associated.

#GO	%dis	sumGOLOR	Description
GO:0001525	4.69	32.13	(BP) angiogenesis
GO:0001871	4.69	32.13	(MF) pattern binding
GO:0003002	4.69	32.13	(BP) regionalization
GO:0005539	4.69	32.13	(MF) glycosaminoglycan binding
GO:0009952	4.69	32.13	(BP) anterior/posterior pattern formation
GO:0030246	4.69	32.13	(MF) carbohydrate binding
GO:0030247	4.69	32.13	(MF) polysaccharide binding
GO:0032388	4.69	32.13	(BP) positive regulation of intracellular transport
GO:0033158	4.69	32.13	(BP) regulation of protein import into nucleus, translocation
GO:0033160	4.69	32.13	(BP) positive regulation of protein import into nucleus, translocation
GO:0042306	4.69	32.13	(BP) regulation of protein import into nucleus
GO:0042562	4.69	32.13	(MF) hormone binding
GO:0045428	4.69	32.13	(BP) regulation of nitric oxide biosynthetic process
GO:0045429	4.69	32.13	(BP) positive regulation of nitric oxide biosynthetic process
GO:0048729	4.69	32.13	(BP) tissue morphogenesis
GO:0051222	4.69	32.13	(BP) positive regulation of protein transport
GO:0090316	4.69	32.13	(BP) positive regulation of intracellular protein transport
GO:0007389	6.25	32.54	(BP) pattern specification process
GO:0010594	6.25	32.54	(BP) regulation of endothelial cell migration
GO:0050431	6.25	32.54	(MF) transforming growth factor beta

			binding
GO:0050679	6.25	32.54	(BP) positive regulation of epithelial cell proliferation
GO:0051896	6.25	32.54	(BP) regulation of protein kinase B signaling cascade
GO:0051897	6.25	32.54	(BP) positive regulation of protein kinase B signaling cascade
GO:0048646	7.81	32.86	(BP) anatomical structure formation involved in morphogenesis

%dis: Percentage of disease genes that are annotated with a given GO term.
